# Supplementary material for: Complete chloroplast genome sequences of Myristicaceae species with the comparative chloroplast genomics and phylogenetic relationships among them
Source: PLoS One. 2023 Mar 20;18(3):e0281042. doi: 10.1371/journal.pone.0281042 (PMC10027215; doi:10.1371/journal.pone.0281042)
Supplement: Attachment — Submitted filename: Response to Reviewers.docx [file pone.0281042.s003.docx]

**Dear Editors and Reviewers:**

Thank you for your letter and for the reviewers’ comments concerning our manuscript entitled “Complete chloroplast genome sequence s of 10 Myristicaceae species with the comparative chloroplast genomics and phylogenetic relationships among them” (ID: PONE-D-22-11654). Those comments are all valuable and very helpful for revising and improving our paper, as well as the important guiding significance to our researches. We have studied comments carefully and have made correction which we hope meet with approval. Revised portion are marked in red in the paper. The revisions were addressed point by point below.

**Response to the journal requirements:**

Journal Requirements:

1. Please ensure that your manuscript meets PLOS ONE's style requirements, including those for file naming. The PLOS ONE style templates can be found at

https://journals.plos.org/plosone/s/file?id=wjVg/PLOSOne_formatting_sample_main_body.pdf and

<https://journals.plos.org/plosone/s/file?id=ba62/PLOSOne_formatting_sample_title_authors_affiliations.pdf>

2. Thank you for stating the following financial disclosure:

 “This work was supported by the National Natural Science Foundation of China (No.31960289)”

Please state what role the funders took in the study.  If the funders had no role, please state: "The funders had no role in study design, data collection and analysis, decision to publish, or preparation of the manuscript."

If this statement is not correct you must amend it as needed.

Please include this amended Role of Funder statement in your cover letter; we will change the online submission form on your behalf.

3. Please include a separate caption for each figure in your manuscript.

**Response:**

1. The manuscript has been modified as required，including those for file naming.

2. This work was supported by the National Natural Science Foundation of China (No.31960289). The funders had no role, and the sentence of “"The funders had no role in study design, data collection and analysis, decision to publish, or preparation of the manuscript." has been added in my cover letter.

3. the separate caption for each figure have listed in the revised manuscript.

**Responds to the reviewer’s comments:**

Reviewer #1:

The authors made a progress to our understanding to the plastome evolution and phylogeny of Myristicaceae. Because of poor support values for the systematic position of H. pandurifolia, you can not make a confident conclusion that Endocomia should be re-established. Detailed discussions on Endocomia should be given, mentioning and comparing studies based on phylogenetic analyses and morphological observations.

A great defect to your study is the neglection of many plastomes of Myristicaceae recently published by Cai et al. (2021) [doi: 10.1111/jse.12556]. More samples would be of great importance to resolve the systematic position of H. pandurifolia and to find new relationships. Additionally, phylogenetic analyses should also combine the data of Myristicaceae provided by PPA II (Li et al. 2021; doi: 10.1186/s12915-021-01166-2].

I also suggest the authors compare your phylogenetic results to Myristicaceae in an updated Chinese vascular plant tree of life (Hu et al. 2020; doi: 10.1111/jse.12642).

In general, I suggest the authors use as many data as possibly, not only yours but also those provided in previous studies.

**Response:**

thanks very much for the valuable and very helpful suggestion about the data phylogeny analysis of Myristicaceae. It is really true as Reviewer suggested that the data in this manuscript was dificient for analysis the phylogenetic of Myristicaceae, especially for *Horsfieldia*. So, we also quoted the conclusions and data of Cai et al. (2021) [doi: 10.1111/jse.12556] to get more reasonable conclusion in the revised paper. At the same time, we mentioning and comparing the morphological observations of the common morphological between *H.pandurifolia* and *Endocomia macrocoma* ssp.*prainii* and the differences between *H.pandurifolia* and other *Horsfieldia* species in revised paper, and discussion the results to the Myristicaceae based on the AFLP analysis ( Wu et al. 2015) [doi:10.7525 /j.issn.1673-5102.2015.05.003]. Additionally, we compared our results to Myristicaceae in an updated Chinese bascular plant tree of life (Hu et al. 2020; doi: 10.1111/jse.12642) in revised paper.

Reviewer #2:

The authors studied the chloroplast genomes of 10 Myristicaceae species, including 2 newly assembled for this study and 8 previously reported, aiming to provide potential genomic resources of this family and molecular evidence for taxonomical revision. The authors contribute to the taxonomy of Myristicaceae using modern technology, bringing novel insights to the science community. The study is very interesting to me. However, before the manuscript is ready for publishing, the following issues must be fixed.

Major issues:

1)The study aimed to provide evidences for the acceptance of the genus Endocomia proposed by W.J. de Wilde who validly published this genus name in 1984. Yet, the manuscript did demonstrate neither in-depth study on the nomenclature and classification of the family Myristicaceae, nor clear presentation on the inconsistency on the morphological description between floras and their own observations. It is not enough by just stating "we found that the character description of a few species was inconsistent with flora records in China as well" (L233-235) in the introduction and "morphological observations of Knema genus by the authors showed that the flowers and leaf of K. cinerea and K. elegans are similar" (L480-481) in the discussion. My recommendation is that the authors should spend more efforts in presenting the inconsistency and similar morphology clearly, which may increase the merit and interests of the study. The authors may also refer to online databases like Tropicos [https://www.tropicos.org/] and Plants of the World Online [https://powo.science.kew.org/] for the validity of scientific names and the current classification preferences, respectively.

2)The study tried to elucidate phylogenetic relationships of Myristacaceae. Yet, vouchering procedures were not clearly shown in the manuscript, particularly Table 1. If you collected specimens for the studied materials, it is a must to present in the manuscript as vouchers. Table 1 should also include collector number, date of collection and deposition (herbarium or institution) for your vouchers. GPS coordinates are recommended to be presented in a single column. A map visualising the distances of collection sites is a bonus. Digitised specimens are recommended to be submitted as supporting information as your proof of authentication.

3) Methodology is incomplete. Methods is missing for dN/dS analysis (i.e. which software you used?) and the authentication of studied materials (which flora(s) you referred to? any personal judgement(s)?).

4) The claims of this study is valid, supported by the clear results of phylogenetic analysis. Horsfieldia pandurifolia exhibited distinct phylogenetic position from the other two sistered Horsfieldia species with great support values. Yet, the introduction scarcely mentioned the application of chloroplast genomes in resolving phylogeny of Myristacaceae AND other angiosperms. Apart from the studies achieved by the authors in the past, the studies of Cai et al. (2021) should be mentioned and discussed. Meanwhile, any phylogenetic studies of Myristacaceae, like the one of Swetha et al. (2019), have been previosly reported? A brief summary is needed.

Cai, C. N., Ma, H., Ci, X. Q., Conran, J. G., & Li, J. (2021). Comparative phylogenetic analyses of Chinese Horsfieldia (Myristicaceae) using complete chloroplast genome sequences. Journal of Systematics and Evolution, 59(3), 504-514.

Swetha, V. P., Sheeja, T. E., & Sasikumar, B. (2019). DNA barcoding to resolve phylogenetic relationship in Myristica spp. Journal of Spices & Aromatic Crops, 28(2).

5) Personally speaking, I think it is a serious problem that the authors did not mentioned the species name of the two newly assembly chloroplast genomes right in the beginnings (abstract and introduction). Meanwhile, I can find no indication on the phylogenetic trees. Such practices negatively affected the clarity of the manuscript, as I do not know which species was newly assembled in this study, which one was previously reported. Simply stating the accession numbers on L276, 307-308 is not clear enough.

Minor issues:

6) Rooms of improvement for the presentation of figures are identified as the followings.

Fig. 3: Missing information as a few characters are invisible as "(trnD-..". The unit of y-axis should be adjusted (should be integer).

Fig. 4: There are many groups of comparisons between species, that the lines and data markers are coloured in similar tones. Frankly speaking, I cannot even read this figure accurately. My suggestion to use distinct colours, and change the shape of data markers instead of solely using dots. For example, use dots as the data markers for H. amygladina vs other species, use triangle for M. yunnanensis vs other species, etc. Fig. 4 should be presented as a single figure with indication (A,B,C) or otherwise three seperated figures. Also, please replace "ration" by "ratio" in the title.

Fig. 5: You should put a line to indicate the threshold of nucleotide diversity as you mentioned in the manuscript. The y-axis must be annotated (Pi). The unit of y-axis should be adjusted (Fig. 5A - 0.001) and (Fig. 5B - 0.005).

Fig. 7: Type I, II & III variation mentioned in the results should be visualised.

Fig. 8: Markers should be put to indicate the two newly assembled chloroplast genomes for this study. Meanwhile, accession number of each genome should be shown together with the species name. The sub-clades mentioned in the results should be visualised.

7) Please be aware that PLOS uses the numbered citation (citation-sequence) method for in-text citation, and the reference style is Vancouver for the reference list.

8) Gene names should be written in italic. Be aware of the presentations in Table 3 and 4.

9) Extensive language proofreading is highly recommended. Be aware of plural and singular forms of nouns, which affects the subsequent form of verbs. Some vocabularies were incorrectly used, conveying different meanings.

Other comments and suggestions for the Authors:

L198-199: … Comparing the genome structure of …

L202 and elsewhere: “sequence divergence analysis” but not “sequences divergence analysis”

L203 and elsewhere: “intergenic spacers” but not “gene spaces” or “gene spacers”

L205-206: … formed a sister clade with Myristica species supported by both high maximum likelihood bootstrap values and Bayesian posterior probabilities;

L219-220: … Since their seeds are rich in C14 fatty acid (Mao et al., 2017) [a space was missed between "acid" and in-text citation]

L233: significant economic opportunity in exploring the resource of Myristaceae species.

L232-233: We conducted a field survey for this family in [region/ provinces] and found that some species appear in small population with few individuals [could you provide an approximate number of individuals?].

L237-239: … platform. To analyze the organization, gene contents, patterns of nucleotide substitution, simple sequence repeats (SSRs), and phylogenetic relationships, the previously reported chloroplast genomes of eight Myristicaceae species were considered for comparative analyses with the two newly assembled chloroplast genomes.

L243: “novel” instead of “abundant”?

L254: suggest to removing the “Note” statement and put in main text

L257: USA). The total genomic DNA per germplasm with over 30 ng μL-1 were used for subsequent steps.

L272-274: … homologous genes in the reference genomes [state the species and accession number here], based on phylogenetic proximity.

L275: the species name for “these species” were clearly present in the revised manuscript.

L285-287: it has been modified to “Performing comparative analysis for the 10 Myristaceae species species, the chloroplast genome of Liriodendron tulipifera (accession number) from the same order Magnoliales were downloaded from the public database NCBI GenBank and used as a reference.”

L289: rearrangement and inverse evolutionary events …

L294 and elsewhere: “coding sequences” instead of “code sequences”

L300-308: This paragraph should be rewritten. MAFFT is used for alignment before constructing ML and BI trees. “Taxonomic positions” instead of “taxonomic locations”. The scientific name should be provided for the eight previously reported chloroplast genomes before the accession number, and they should be mentioned in the beginning of the manuscript.

L312-313 and elsewhere: quadripartite structure typical of angiosperms

L313-314: please check the size of the largest LSC, I found 92,561 bp in table 1

L314: 48,154 bp

L318-319: The number of genes ranged from 121 to 131, with varied numbers …

L319: suggest deleting “unique”

L320: suggest deleting “-fragment”

L325: K. conferta and (a space between "conferta" and “and” was missed)

L325-326: K. globularia should be italic

L326: Gene with copies? The number of copy (i.e. 1,2,3) should be clearly indicated in this table.

L335 and elsewhere: “the studied species” instead of “all species”

L338: please double check Table 4. I cannot find hexanucleotide (AATAAA)3 for H. pandurifolia in table 4, but H. kingii. If necessary, please revise the presentation of the table e.g. adding cell borders.

L342: genew??

L342,345,346: suggest replacing “genes or intergenic regions” by “in”.

L342 and elsewhere: “IR regions” instead of “IR region”

L346-347: “Among them” instead of “In these sequences”

L348: …, while the longest TRS …

L352: suggest removing the “Note” statement. If feasible, you may choose to show the information of mono- and di-nucleotides SSRs sites in supporting information.

L357,359,361,363 and elsewhere talking about “Sequence Divergence”: “PCGs” instead of “genes”

L363: “Most PCGs” instead of “Some genes”

L367: we calculated the nucleotide diversity (Pi) values of the PCGs and intergenic spacers.

L372-375 and elsewhere: you used “~” to present intergenic spacers in previous paragraphes. Please keep consistency. Choose either “~” or “-”.

L379: …, and the 3 termination codons …

L384: … Myristicaceae were relatively conserved.

L385 and elsewhere: “inverted repeat regions” but not “reverse repeat region”

L386: you already abbreviated “protein coding genes” as “PCGs”. Please use the abbreviation here.

L387: "highly" instead of “more highly”

L397: SSC regions. Except H. pandurifolia, the studied Myristicaceae species had the same set of genes at the border: the rpl22 …

L400: please add a description on the differences of H. pandurifolia here.

L400: what kind of “variation”? Any range?

L402: “LSC/IRs” but not “LSC/IRb”, since rps19 was also find in the border “LSC/IRa”

L405 and elsewhere: “phylogenetic trees” but not “phylogenetic tree”, since you have both ML and BI trees.

L409: sub-clade 1 should be marked in Fig. 8

L411: In the sub-clade 2, …

L413: “source plants” but not “producers”

L414: In-text citation is needed for this sentence.

L420: “consisted of” but not “included”

L422: … Compared to the species from the same order Magnoliales,

L423: “were” not “was”

L428: …, and the results are consistent with previous studies that most SSRs were

L429: … were located in ycf genes

L430: delete “, consistent with other research”

L434 and elsewhere: “previous studies” instead of “other research”

L436: “majority of” but not “major”

L443-446: please double check this sentence. Is the reason correct?

L446: Type I, II and III should be visualised in Fig. 7

L447-448: … LSC and SSC regions, respectively. In contrast, only …

L448: I cannot see “only the gene ycf1 was near the border” in Fig. 7

L449-452: except H. pandurifolia, that the two rps19 were located in the IR regions. Type III was found in all the studied species, except H. pandurifolia, that trnH was located in the LSC region whereas trnH was located in IR region of H. pandurifolia.

L458: what is the results reported in other species? Please describe.

L460: In-text citation is needed for the sentence “… estimate evolutionary relationships”.

L460-462: …, DNA barcoding have been considered as a reliable tool for resolving phylogenetic relationships and species authentication.

L464: “mutation hotspots” but not “genes and gene spacers”

L465: phylogenetic relationships

L466-467: Comparative genome analysis using mVISTA showed that the genomes are relatively conserved with minor variation, …

L468-469: …, there were no considerable rearrangement being detected in the chloroplast genomes.

L470: “phylogenetic relationships” but not “phylogenetic analysis”

L472: … 2015). In this study, the…

L473: “phylogenetic relationships” but not “phylogenetic tree”

L474 and elsewhere: “The genus XXX” but not “The XXX genus”

L476-477: … separated from the two Horsfieldia species forming a single clade.

L479-480: this study suggests taxonomical revision that H. pandurifolia should be separated from the genus Horsfieldia and placed in the genus Endocomia (1984) proposed by W.J. de Wilde [in-text citation of the protolgue is needed here].

L481: “leaves” but not “leaf”

L486: In this study, we sequenced two chloroplast genomes of Knema species, [species names needed], and compared them with those of eight reported species.

L487: "genomes" but not "genome"

L489: how many of “some”? Please provide a number instead of just saying “some”.

L489: “explored” instead of “developed”

L493-495: … a single clade, suggesting taxonomical revision that H. pandurifolia should be separated from Horsfieldia and placed in the genus Endocomia.

**Response:**

**Response for major issue:**

1)We have re-written the part of classification of Myristicaceae, including “introduction” and “discussion” . we described the phylogenetic location of *H. pandurifolia* currently reported in detail, and compared the different morphological characteristics found in the actual investigation to the flora of China. At the same time, we mentioning and comparing the morphological observations of the common morphological between *H.pandurifolia* and *Endocomia macrocoma* ssp.*prainii* and the differences between *H.pandurifolia* and other *Horsfieldia* species in revised paper, and the same discussion was performed between *K. cinerea* and *K. elegans*.

For the validity of scientific names and the current classification preferences, we have modified the species names refer to the databases like Tropicos and Plants of the World Online.

2) accept the reviewer’s comments, the revisions and explain are below:

all materials used in this study were kept in situ in nature reserves or planted in Yunnan Institute of Tropical Crops, so, the isolated specimens were not made. The collector number,date of collection and planting location were listed in Table 1 in revised manuscript. GPS coordinates were presented in single column, and a map visualising the distances of collection sites is showed in revised manuscript.

3) “the DnaSP software was used in analysis the dN and dS of all species in this study”, this sentence has been added in the “Methods” section in the revised manuscript.

We are very sorry for our negligence to explain the authentication of studied materials. When collected the materials used in this study ,the species were identified according to the records in Flora of China, this modification is added in the revised manuscript.

4) in revised manuscript, it has been added in the introduction section about the application of chloroplast genomes in resolving phylogeny of Myristicaceae and other angiosperms. mentioned and discussed the previous report of Cai et al and Swetha et al about Myristicaceae.

5) It is indeed our negligence that the two newly assembly chloroplast genomes did not mentioned in the abstract and introduction. We have added the two newly assembly chloroplast name in the abstract and introduction section, and indicated the two newly assembly chloroplast genomes on the phylogenetic trees in the revised manuscript.

**Response for minor issue:**

6)We have modified the “Fig. 3” by the reviewer’ suggestion. Due to adding a figure (Fig. 1) in manuscript, “Fig. 4” instead of original “ Fig. 3”

Fig. 4 is really complicated, according to the reviewer’s suggestion to use distinct colors, and change the shape of data markers, but it still not well distinguish the data of different groups, many points are overlapping, so avoid confusion, the author will upload the value table namely **S1** **Table**, it is more conducive to find each group of dS, dN and dN / dS values, so the figure of dS and dN / dS were deleted. In order to visually check the genes of dN / dS > 1 in this study, only the figure of dN / dS > 1 was retained in the revised manuscript. Due to adding a figure (Fig. 1) in manuscript, “Fig. 5” instead of original “ Fig. 4”

Fig. 5: the threshold of nucleotide diversity has been added and the y-axis has been annotated (Pi) in the revised manuscript. Because of the range of Pi value among Fig.5A and Fig. 5B was different, so we can not adjust the unit of y-axis. Due to adding a figure (Fig. 1) in manuscript, “Fig. 6” instead of original “ Fig. 5”

Fig. 7: No. Type I, II & III means the gene model near the LSC,SSC and IR border in the studied species. It has been described in text and not visualized in Fig. 7, because that would make Fig. 7 complicated. Due to adding a figure (Fig. 1) in manuscript, “Fig. 8” instead of original “ Fig. 9”

Fig. 8: the two newly assembled chloroplast genomes for this study have been Marked using blue font. Meanwhile, accession number of each genome have been shown together with the species name. The sub-clades mentioned in the results have been visualised using “sub-cladeⅠ” and “sub-cladeⅡ” in revised manuscript. Due to adding a figure (Fig. 1) in manuscript, “Fig. 9” instead of original “ Fig. 8”

7) It has modified according the PLOS requirement.
8) The gene names have been written in italic in the revised manuscript.
9) We are sorry for the lack of language expression. The plural and singular forms of nouns have been checked and corrected. The language of revised manuscript has been checked carefully again.

**Response to other comments and suggestions:**

L198-199: it has been modified to “Comparing the genome structure of …”

L202 : “sequences divergence analysis” has been modified to “sequence divergence analysis”

L203: “gene spaces” or “gene spacers” has been modified to “intergenic spacers”

L205-206: it has been modified to “… formed a sister clade with Myristica species supported by both high maximum likelihood bootstrap values and Bayesian posterior probabilities”

L219-220: it has been modified to “… Since their seeds are rich in C14 fatty acid (Mao et al., 2017)”

L222: “implying a significant economic opportunity in developing these species” has been modified to “significant economic opportunity in exploring the resource of Myristaceae species”.

L232-233: We conducted a field survey for this family in [region/ provinces] and found that some species appear in small population with few individuals [could you provide an approximate number of individuals?].

L237-239: it has been modified to “To analyze the organization, gene contents, patterns of nucleotide substitution, simple sequence repeats (SSRs), and phylogenetic relationships, the previously reported chloroplast genomes of eight Myristicaceae species were considered for comparative analyses with the two newly assembled chloroplast genomes.”

L243: the “novel” is better than “abundant”, it has been modified.

L254: the “Note” has been removed and put in main text

L257: it has been modified to “The total genomic DNA per germplasm with over 30 ng μL-1 were used for subsequent steps.”

L272-274: the species and accession number have been added in the revised manuscript.

L275: the name of “these species” have been added.

L285-287: it has been modified to “ Performing comparative analysis for the 10 Myristaceae species species, the chloroplast genome of Liriodendron tulipifera (NC_008326.1) from the same order Magnoliales were downloaded from the public database NCBI GenBank and used as a reference.”

L289: it has been modified to “ rearrangement and inverse evolutionary events …”

L294 it has been modified to “coding sequences” .

L300-308: This paragraph have been rewritten. “taxonomic locations” has modified to “Taxonomic positions”. The accession number of eight previously reported chloroplast genomes have mentioned in “Materials and Methods”, and they have been listed in the Table 1.

L312-313 it has been modified to “quadripartite structure typical of angiosperms”

L313-314: it has been modified to 92,561 bp.

L314: it has been modified “48,154 bp”

L318-319: it has been modified to “The number of genes ranged from 121 to 131, with varied numbers”

L319:“unique” has been deleted

L320: “-fragment” has been deleted

L325: “K. confertaand” is a mistake, it has been modified to “K. conferta,”

L325-326: K. globularia has modified to “ *K. globularia*”

L326: Gene with copies? The number of copy (i.e. 1,2,3) should be clearly indicated in this table.

L335 it has been modified to “the studied species”

L338: due to a missing table border, the repeat units between species are not well distinguished. The table has been adjusted with adding cell borders. The hexanucleotide (AATAAA)3 is really belongs to *H. pandurifolia*.

L342: it is “genes” and it has been replaced by “in”.

L342,345,346:it has been modified to “in”.

L342: it has been modified to “IR regions”

L346-347: it has been modified to “Among them”

L348: it has been modified to “…, while the longest TRS …”

L352: the mono- and di-nucleotides SSRs sites were listed in S1 Table. The “Note” statement has been removed.

L357,359,361,363: it has been modified to “PCGs”

L363: it has been modified to “Most PCGs”

L367: it has been modified to “we calculated the nucleotide diversity (Pi) values of the PCGs and intergenic spacers”

L372-375: it has been modified to “~”

L379: it has been modified to “…, and the 3 termination codons …”

L384: it has been modified to “… Myristicaceae were relatively conserved.”

L385: it has been modified to “inverted repeat regions”

L386: it has been abbreviated as “PCGs”

L387: it has been modified to "highly"

L397: it has been modified to “SSC regions. Except *H. pandurifolia*, the studied Myristicaceae species had the same set of genes at the border: the rpl22 …”

L400: the differences of *H. pandurifolia* have been added

L400: this sentence means the length variation of chloroplast genome of studied species. The deletion of this sentence does not affect the result of whole article, so it has been deleted in the revised manuscript

L402: it has been modified to “LSC/IRs”

L405: it has been modified to “phylogenetic trees”

L409: it has been marked “sub-clade 1” and “sub-cladeⅡ” in Fig. 8

L411: it has been modified to “In the sub-clade 2, …”

L413: it has been modified to “source plants”

L414: the citation was added in the revised manuscript

L420: it has been modified to “consisted of”

L422: it has been modified to”… Compared to the species from the same order Magnoliales,”

L423: it has been modified to “were”

L428: it has been modified to “…, and the results are consistent with previous studies that most SSRs were”

L429: it has been modified to “… were located in ycf genes”

L430: “, consistent with other research” has been deleted

L434: it has been modified to“previous studies”

L436: it has been modified to “majority of”

L443-446: through check again, it is correct

L446: in the text, the sentence represents three types of the border, If it is visualized, Fig. 7 appears confusing and was not modified in the manuscript

L447-448: it has been modified to “… LSC and SSC regions, respectively. In contrast, only …”

L448: this sentence was deleted because of the mistake statistics

L449-452: it has been modified to “except *H. pandurifolia*, that the two rps19 were located in the IR regions. Type III was found in all the studied species, except *H. pandurifolia*, that trnH was located in the LSC region whereas *trnH* was located in IR region of *H. pandurifolia*.”

L458: it has been modified in the revised manuscript

L460: the citation was added in the revised manuscript

L460-462: it has been modified to “…, DNA barcoding have been considered as a reliable tool for resolving phylogenetic relationships and species authentication.”

L464: it has been modified to “mutation hotspots”

L465: it has been modified to “phylogenetic relationships”

L466-467: it has been modified to “Comparative genome analysis using mVISTA showed that the genomes are relatively conserved with minor variation, …”

L468-469: it has been modified to “…, there were no considerable rearrangement being detected in the chloroplast genomes.”

L470: it has been modified to “phylogenetic relationships”

L472: it has been modified to ‘’… 2015). In this study, the…”

L473: it has been modified to “phylogenetic relationships”

L474 : it has been modified to“The genus XXX”

L476-477: it has been modified to “… separated from the two *Horsfieldia* species forming a single clade.”

L479-480: it has been modified to “this study suggests taxonomical revision that *H. pandurifolia* should be separated from the genus *Horsfieldia* and placed in the genus *Endocomia* (1984) proposed by W.J. de Wilde” and added the citation in the revised manuscript.

L481: it has been modified to “leaves”

L486: it has been modified to “In this study, we sequenced two chloroplast genomes of Knema species, *K. globularia* and *K. cinerea*, and compared them with those of eight reported species.”

L487: it has been modified to "genomes"

L489: it is “eleven highly variable sites” and modified in the revised manuscript.

L489: it has been modified to “explored”

L493-495: it has been modified to “… a single clade, suggesting taxonomical revision that *H. pandurifolia* should be separated from *Horsfieldia* and placed in the genus *Endocomia*.”
