# Supplementary material for: Complete chloroplast genome sequences of Myristicaceae species with the comparative chloroplast genomics and phylogenetic relationships among them
Source: PLoS One. 2023 Mar 20;18(3):e0281042. doi: 10.1371/journal.pone.0281042 (PMC10027215; doi:10.1371/journal.pone.0281042)
Supplement: Attachment — Submitted filename: Response to Reviewers.docx [file pone.0281042.s004.docx]

**Dear Editors and Reviewers:**

Thank you for your letter and for the reviewers’ comments concerning our manuscript entitled “Complete chloroplast genome sequences of Myristicaceae species with the comparative chloroplast genomics and phylogenetic relationships among them” (ID: PONE-D-22-11654R1). Those comments are all valuable and very helpful for revising and improving our paper, as well as the important guiding significance to our researches. We have studied comments carefully and have made correction which we hope meet with approval. Revised portion are marked in red in the paper. The revisions were addressed point by point below.

**Response to the journal requirements:**

Journal Requirements:

The reviewers have additional suggestions/concerns regarding the revised submission. Reviewer 1 does not think that their criticisms were adequately addressed. They still has concerns regarding the addition of samples to the plastid phylogeny to provide a more holistic view of the relationships within the study system. Reviewer 2 outlined more minor issues that should be addressed.

Additionally, I ask you to include greater detail regarding the methods. The version numbers of all software used should be indicated; full details regarding the parameters used in each analysis should be included; and the data should be deposited in publicly accessible repositories. Much more information is require to reproduce the analyses presented here.

Please include the following items when submitting your revised manuscript:

- A rebuttal letter that responds to each point raised by the academic editor and reviewer(s). You should upload this letter as a separate file labeled 'Response to Reviewers'.
- A marked-up copy of your manuscript that highlights changes made to the original version. You should upload this as a separate file labeled 'Revised Manuscript with Track Changes'.
- An unmarked version of your revised paper without tracked changes. You should upload this as a separate file labeled 'Manuscript'.

**Response:**

1. The data of Cai et al. proposed by reviewer 1 have been added to analysis the phylogenetic relationships of Myristicaceae, and the minor issues proposed by reviewer 2 has been modified in the revised manuscript.

2. the details about methods were added to the revised manuscript, including parameter settings and software version, but some online analysis software only needs to upload the data to analyze with no special requirements, therefore, in the revised manuscript, the website of online analysis software was added in the part of “reference”, and detailed operation instructions of these software were not conducted in the part of “materials and methods”. The data of this study have been deposited in the GenBank public database.

3.Including “Responds to Reviewer’s” “Revised Manuscript with Track Changes” and “Manuscript” files when submitting our revised manuscript.

**Responds to the reviewer’s comments:**

Reviewer #1:

 I considered there is no significant improvement for our undestanding on the plastid phylogeny of Myristicaceae. One of the main findings of this study that Horsfieldia pandurifolia should be treated as Endocomia macrocoma subsp. prainii is not found in this study for the first time, but has been widely accepted in recent twenty years.

The authors did not follow my suggestions to combine more available data from Cai et al. (2021) and Li et al. (2021) for phylogenetic analyses.

**Response:**

The classification position of *Horsfieldia pandurifolia* in the Myristiaceae has been controversial. *H. pandurifolia* was first named in 1963, and named *Endocomia macrocoma* ssp. *prainii* by de Wilde in 1984; Ye again named *Horsfieldia macrocoma* in 2004*,* in the Flora of China published in 2008, this species was named *Horsfieldia prainii*, Wu identify with de Wilde's suggestion that this species should named *Endocomia macrocoma* ssp. p*rainii* in 2015, Cai suggest that this species belong to genus *Horsfieldia* in 2019. So, the taxonomic position of this species has been debated in the genera *Horsfieldia* and *Endocomia*, our study mainly focuses on the taxonomic location of *H. pandurifolia* in Myristicaceae. If the name of *H. pandurifolia* widely accepted, it would not have debated. Therefore, we only suggested the taxonomic position and naming of the species in Myristicaceae based on the results of this study, and the name has been proposed by the previous researchers, not to renaming it.

It is a very good suggestion that combine more available data to perform the phylogenetic analyze. In the revised manuscript, based on the “PPAⅡ” tree from Li et al (2021), we select *Liriodendron tulipifera* as the outgroup and combine the data from Cai et al.(2021) to analysis the phylogenetic relationships of Myristicaceae. The modification have added in the revised manuscript.

Reviewer #2:

The authors made a great progress on improving the manuscript, adequately addressing my comments. Yet the author could consider the following suggestions in further polishing their manuscript:

L58: "considered a high-quality raw material [3-6], implying a significant economic opportunity in"

L110-L113: I suggest to revise the sentence on colllection sites and specimens as "The collection sites included Nangunhe National Nature Reserve, Xishuangbanna National Nature Reserve and Yunnan Institute of Tropical Crops (YITC), Yunnan, China. All the live specimen was planting on site."

L139: "..., the accession numbers were ... "

L161: "..., DnaSP software ..."

L204: "and rpl32~trnL-UAG in the SSC. Among them, ..."

L259-260: "To determine the phylogenetic relationship of the 10 species of Myristicaceae, phylogenetic trees were reconstructed ..."

L270: The citation (http://www.plantplus.cn/rep/protlist) should be properly cited in Vancouver style!!!

L284: "regions, followed by coding regions, and most TRSs were located in ycf genes and non-coding"

L324-325: "... In this study, the phylogenetic relationships of 10 Myristicaceae species were inferred using ML and Bayesian methods."

Table 1 and elsewhere: please carefully check the accession numbers of your chloroplast genomes. The one of Knema cinerea (Poir.) Warb. should be "MN683756" but not "MN583756"!

**Response:**

L58: it has been modified to "considered a high-quality raw material [3-6], implying a significant economic opportunity in" in the revised manuscript.

L110-L113: it has been modified to "The collection sites included Nangunhe National Nature Reserve, Xishuangbanna National Nature Reserve and Yunnan Institute of Tropical Crops (YITC), Yunnan, China. All the live specimen was planting on site."

L139: it has been modified to "..., the accession numbers were ... "

L161: it has been modified to "..., DnaSP software ..."

L204: it has been modified to "and rpl32~trnL-UAG in the SSC. Among them, ..."

L259-260: it has been modified to "To determine the phylogenetic relationship of the 10 species of Myristicaceae, phylogenetic trees were reconstructed ..."

L270: The “http://www.plantplus.cn/rep/protlist” has cited in Vancouver style in the revised manuscript

L284: it has been modified to "regions, followed by coding regions, and most TRSs were located in ycf genes and non-coding"

L324-325: it has been modified to "... In this study, the phylogenetic relationships of 10 Myristicaceae species were inferred using ML and Bayesian methods."

Table 1 and elsewhere: it has been modified to "MN683756"
